# Effect of Direct Electrical Current on Bones Infected with *Staphylococcus epidermidis*

**DOI:** 10.1002/jbm4.10119

**Published:** 2019-01-31

**Authors:** Suzannah M Schmidt‐Malan, Cassandra L Brinkman, Melissa J Karau, Robert A Brown, Brian E Waletzki, Lawrence J Berglund, Audrey N Schuetz, Kerryl E Greenwood‐Quaintance, Jayawant N Mandrekar, Robin Patel

**Affiliations:** ^1^ Division of Clinical Microbiology Department of Laboratory Medicine and Pathology Mayo Clinic Rochester MN USA; ^2^ Biomaterials and Histomorphometry Core Laboratory Mayo Clinic Rochester MN USA; ^3^ Biomechanics Research Core Laboratory Mayo Clinic Rochester MN USA; ^4^ Division of Biomedical Statistics and Informatics Department of Health Sciences Research Mayo Clinic Rochester MN USA; ^5^ Division of Infectious Diseases Department of Medicine Mayo Clinic Rochester MN USA

**Keywords:** ELECTRICAL CURRENT, BIOFILM, IMPLANT INFECTION, ANTIBIOFILM ACTIVITY

## Abstract

We are developing electrical approaches to treat biofilm‐associated orthopedic foreign‐body infection. Although we have previously shown that such approaches have antibiofilm activity, the effects on bone have not been assessed. Herein, low‐amperage 200 μA fixed direct current (DC) was compared with no current, in a rat femoral foreign‐body infection model. In the infected group, a platinum implant seeded with *S. epidermidis* biofilm (10^5^ CFU/cm^2^), plus 50 μL of a 10^9^ CFU suspension of bacteria, were placed in the femoral medullary cavity of 71 rats. One week later, rats were assigned to one of four groups: infected with no current or DC, or uninfected with no current or DC. After 2 weeks, bones were removed and subjected to histopathology, micro‐computed tomography (μCT), and strength testing. Histopathology showed no inflammation or bony changes/remodeling in the uninfected no current group, and some osteoid formation in the DC group; bones from the infected no current group had evidence of inflammation without bony changes/remodeling; along with inflammation, there was moderate osteoid present in the DC group. μCT showed more cortical bone volume and density, trabecular thickness, and cancellous bone volume in the DC group compared with the no current group, for both uninfected and infected bones (*p* < 0.05). There was no difference in torsional strength or stiffness between the no current versus DC groups, for both infected and uninfected bones (*p* > 0.05). © 2018 The Authors. *JBMR Plus* Published by Wiley Periodicals, Inc. on behalf of the American Society for Bone and Mineral Research.

## Introduction

Joint replacement surgery is increasingly common in the United States.^1^ However, with the increase in arthroplasties comes an increased chance of prosthetic joint infection (PJI). Approximately 2% of hip and knee joints become infected,^2^, ^3^, ^4^ with 30% and 23% of hip and knee PJIs, respectively, attributed to the coagulase‐negative *Staphylococcus* species. *Staphylococcus epidermidis* is the predominant cause of PJIs.^1^

Because organisms associated with PJI are typically found in biofilms, PJI is difficult to treat with antibiotics alone.^1^, ^5^, ^6^ We have previously described the use of low‐amperage fixed direct current (DC) as an antibiofilm strategy in *in vitro* and *in vivo* rabbit and rat foreign‐body osteomyelitis models, showing that amperages as low as 2 μA *in vitro* and 200 μA *in vivo* reduced bacterial loads compared with untreated controls.^7^, ^8^, ^9^, ^10^ In addition, other investigators have shown that *S. epidermidis* biofilms can be made to detach from surgical stainless steel by the use of DC of 100 μA.^11^, ^12^ Up until now, however, few studies on the effects of DC on bone formation/resorption and strength have been performed.

Here, strength testing (torsional strength and stiffness) and micro‐computed tomography (μCT—used to measure cortical and cancellous bone volume and density and trabecular separation and thickness) were applied as indices of bone flexibility and strength. Pathology was employed to assess adverse effects at the tissue level, as well as bone formation and resorption, using bones that were uninfected or infected with *S. epidermidis* that had received 200 μA DC or no current.

## Materials and Methods

### Microorganism

*S. epidermidis* Xen 43 (a kind gift of Xenogen Corp., Hopkinton, MA, USA, now Caliper Life Sciences), a bioluminescent strain derived from the parental strain *S. epidermidis* 1457 (a clinical isolate able to form *in vivo* biofilms), was studied. The isolate was passaged through a rat bone and saved in a Microbank vial (Pro‐Lab Diagnostics, Round Rock, TX, USA) at −80°C.

### Experimental rat model

**Figure 1:**
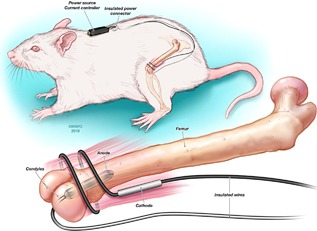
Graphic of rat model and placement of wires and battery pack.^8^

This study was approved by the Mayo Clinic Institutional Animal Care and Use Committee. A modified foreign‐body osteomyelitis model was established in male Wistar rats (Envigo, Huntingdon, Cambridgeshire, UK), weighing 300 g, as done previously,^8^ in which 50 μL of a 10^9^ CFU suspension of *S. epidermidis* was injected into the medullary cavity of the left femur, followed by implantation of a 10‐ × 3‐mm platinum wire preseeded with *S. epidermidis* biofilm (approximately 10^5^ CFU/cm^2^) by incubation in trypticase soy broth (TSB) with 10^6^ CFU/mL *S. epidermidis* for 2 hours. The platinum wire acted as the foreign body and also served as an anode that was connected to an insulated power cable that exited the bone via the bone defect. The cathode was an uninsulated 0.5‐ (outside diameter) × 25‐mm (length) stainless steel wire surgically wrapped around the femur and muscle and connected to an insulated power cable. The power cables concluded with an insulated power connector (Fig. 1). For the duration of the experiment, animals were maintained singly (because of the likelihood of cage mates chewing on wires and jackets) in standard housing with corn bedding and free access to water and standard chow, on a 12‐hour light/dark cycle with weekly enrichment.

### Infected study group

**Figure 2:**
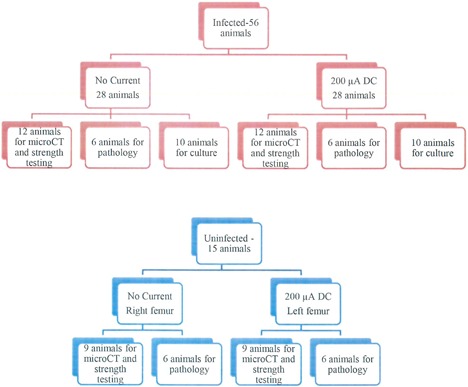
Diagram outlining the distribution of the animal groups studied.

One week after establishing infection, treatment was initiated. Fifty‐six animals were randomly assigned to one of two study arms: no current (*n* = 28) or 200 μA DC (*n* = 28) (Fig. 2). Battery packs (which consisted of a battery and electronic circuitry to regulate the electrical circuit delivered to the electrodes) programmed to deliver 200 μA DC, were attached to the external wires (Fig. 1). Fixed DC was administered for 14 days. Rats were euthanized on day 14 with CO_2_ inhalation. Of the 56 animals, 10 no current and 10 200 μA DC left femurs were aseptically removed, frozen to −80°C, and used for culture. Bone surrounding the implanted wire was cut (a 5 mm section), weighed, and refrozen at −80°C, then pulverized for quantitative bacterial culture. Crushed bone was placed in 2 mL of TSB, vortexed for 30 s, sonicated at 40 kHz for 5 min, vortexed for 30 s, serially diluted, and plated on trypticase soy agar plates containing 5% sheep blood (TSA II; Becton Dickinson, Franklin Lakes, NJ, USA). The wire was removed from the bone and placed in 1 mL of TSB and cultured, as described above. Quantitative culture results for bone and wire were obtained after 48 hours of incubation at 37°C and expressed as log_10_ CFU/g or log_10_ CFU/cm^2^, respectively. For the remaining 36 animals, left femurs were aseptically removed and placed in saline‐soaked gauze. The femurs were frozen at −80°C.

### Uninfected study group

Fifteen animals had anodes and cathodes placed in the left femur as described above, but were not infected (Fig. 2). Battery packs were attached 1 week after placement of anodes and cathodes, and delivered 200 μA DC. Electricity was administered for 14 days. Rats were euthanized with CO_2_ inhalation; the left femur was aseptically removed and placed in saline‐soaked gauze. The right femur was also removed and placed in saline‐soaked gauze to serve as the uninfected no current bones. The femurs were frozen at −80°C.

### Micro‐computed tomography

Twenty‐four infected left femurs (12 no current and 12 200 μA DC) and 18 uninfected femurs (9 right no current and 9 left 200 μA DC) underwent μCT in a Bruker SkyScan‐1272 (Micro Photonics, Allentown, PA, USA; Fig. 2). For imaging, the platinum electrodes were left in place, as removal of the electrodes would have caused the bones to break. Because the electrodes were left in place, the scatter from the μCT made it challenging to analyze the full length of the bone containing the electrode; therefore, a defined region of interest (ROI) was used for each image and analysis. The ROI started approximately 500 μm below the implant, and from that spot on, a ROI of 1000 μm was measured. Within the ROI, 61 projections (each approximately 16.3 μm in thickness) were taken. Of these projections, the cortical bone volume and density (bone surface/tissue volume ratio), as well as the cancellous bone thickness, separation, volume, and density (bone surface/tissue volume ratio), were measured according the the guidelines for the assessment of bone microstructure in rodents using μCT.^13^ These measurements were used as indices of bone strength; for instance, if the cortical and cancellous bone volume and densities were lower in the treated group compared with controls, the bones would be considered to have a higher probability of weakness/fracture. If the trabecular thickness was lower and trabecular separation was higher in the treated versus control groups, this would also mean the bone would be considered to have a higher probability of weakness.

### Strength testing

The same 42 femurs that underwent μCT were used for strength testing. Specimens were carefully cleaned and the distal and proximal ends were potted with methyl methacrylate into ½‐ × ½‐ × ½‐inch polycarbonate tubing aligned, with the help of an alignment jig, such that the long axis of the femur was in the center axis of both adapters. The bones were placed into a custom‐built biaxial electromechanical test device, using a low‐capacity (inch‐ounce) reaction torque sensor RTS‐200 (Transducer Techniques, Temecula, CA, USA). Fixtures on the test device held the samples for torsion testing at 30 degrees/s. Two flexible helical couplings were used to reduce bending stresses on the samples, except for torsion. The torsional strength and stiffness were measured, strength being the ability of the bones to withstand a twisting load (flexibility), and stiffness being the torque per radian twist (eg, if two femurs had the same geometry, but one was weaker because it had less density, it would be less stiff).

### Pathology

Twelve infected left femurs (6 no current, and 6 200 μA DC) and 12 uninfected femurs (6 right no current, and 6 left 200 μA DC) (Fig. 2) were embedded in methyl methacrylate, sectioned longitudinally, and stained with H&E and trichrome. Slides were analyzed for evidence of infection, possible damage to bones from the supplied current, and bone formation/resorption.

### Statistical methods

Statistical analyses were performed using SAS software (SAS Institute, Inc., Cary, NC, USA). Sample sizes of groups were derived to reach 80% power, with at least 1 SD, with a 5% level of significance. Using the Wilcoxon rank‐sum test, we compared the log_10_ CFU/g of bone for the no current and 200 μA DC groups. We also compared the μCT measurements and strength‐testing results for the uninfected no current and 200 μA DC groups as well as the infected no current and 200 μA DC groups. All tests were two‐sided; *p* values of <0.05 were considered statistically significant.

## Results

### Culture

**Figure 3:**
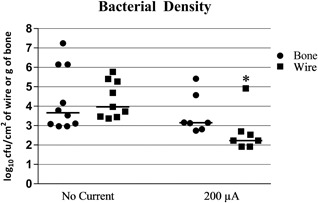
Bacterial density after exposure to no current or 200 μA fixed direct electrical current in foreign body osteomyelitis caused by *Staphylococcus epidermidis* Xen 43.*Significant compared with no current. Bar represents median value.

The median bacterial quantity on the wires receiving 200 μA DC was 2.23 (range 1.92 to 4.92) log_10_ CFU/cm^2^, which was statistically significantly reduced compared with wires receiving no current (median, 3.97; range, 3.37 to 5.77; *p* = 0.0069) (Fig. 3). The median bacterial quantity in the bones receiving 200 μA DC current was 3.15 (range, 2.74 to 5.42) log_10_ CFU/g of bone versus 3.66 (range, 2.97 to 7.24) log_10_ CFU/g of bone for the bones receiving no current (*p* = 0.3792) (Fig. 3).

### Micro‐computed tomography

#### Cortical bone volume

**Figure 4:**
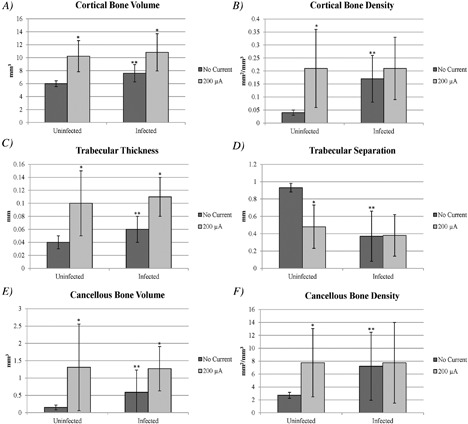
Mean with ± SD μCT measurements of infected and uninfected bones receiving 200 μA fixed direct electrical current or no current: (*A*) cortical volume, (*B*) cortical bone density, (*C*) trabecular thickness, (*D*) trabecular separation, (*E*) cancellous bone volume, and (*F*) cancellous bone density. *Significant compared with no current within infected or within uninfected groups. **Significant compared with no current between infected and uninfected groups.

Cortical bone volume results are shown in Fig. 4 *A*. The median cortical bone volume in the infected group receiving no current was 7.20 (range, 6.03 to 9.83) mm^3^, and was 10.91 (range, 7.30 to 17.62) mm^3^ in the infected group receiving 200 μA DC (*p* = 0.0034). In the uninfected groups, the median cortical bone volume of those receiving no current was 5.75 (range, 5.56 to 6.98) mm^3^, and in the 200 μA DC group the median was 10.09 (range, 6.05 to 13.43) mm^3^ (*p* = 0.0009). When comparing the cortical bone volume of the infected versus uninfected groups receiving no current, the infected group had more bone volume than the uninfected group (*p* = 0.0016). There was no difference in cortical bone volume when comparing the 200 μA DC infected versus uninfected groups (*p* = 0.9648).

#### Cortical bone density

Cortical bone density (bone surface/tissue volume; BS/TV) results are shown in Fig. 4 *B*. The median cortical bone density in the infected group receiving no current was 0.16 (range, 0.05 to 0.36) mm^2^/mm^3^, and was 0.16 (range, 0.09 to 0.46) mm^2^/mm^3^ in the 200 μA DC infected group (*p* = 0.5688). In the uninfected groups, the median cortical bone density of the group receiving no current was 0.05 (range, 0.01 to 0.05) mm^2^/mm^3^, and was 0.15 (range, 0.05 to 0.41) mm^2^/mm^3^ in the 200 μA DC group (*p* = 0.0023). When comparing the cortical bone density of the infected versus uninfected groups receiving no current, the infected group had more bone density than the uninfected group (*p* = 0.0002). There was no difference in cortical bone density when comparing the 200 μA DC infected versus uninfected groups (*p* = 0.5660).

#### Trabecular thickness

Trabecular thickness results are shown in Fig. 4 *C*. The median trabecular thickness in the infected group receiving no current was 0.06 (range, 0.04 to 0.11) mm, and was 0.10 (range, 0.09 to 0.16) mm in the 200 μA DC infected group (*p* = 0.0016). In the uninfected groups, the median trabecular thickness of the group receiving no current was 0.04 (range, 0.04 to 0.08) mm, and was 0.08 (range, 0.05 to 0.17) mm in the 200 μA DC group (*p *= 0.0017). When comparing the trabecular thickness of the infected versus uninfected groups receiving no current, the infected group had more thickness than the uninfected group (*p* = 0.0135). There was no difference in trabecular thickness when comparing the 200 μA DC infected versus uninfected groups (*p* = 0.2004).

#### Trabecular separation

Trabecular separation results are shown in Fig. 4 *D*. The median trabecular separation in the infected group receiving no current was 0.19 (range, 0.12 to 0.95) mm, and was 0.37 (range, 0.10 to 0.81) mm in the 200 μA DC infected group (*p* = 0.8494). In the uninfected groups, the median trabecular separation of the group receiving no current was 0.95 (range, 0.81 to 0.96) mm, and was 0.57 (range 0.13 to 0.84) mm in the 200 μA DC group (*p* = 0.0005). When comparing the trabecular separation of the infected versus uninfected groups receiving no current, the uninfected group had significantly more separation than the infected group (*p* = 0.0005). There was no difference in trabecular separation when comparing the 200 μA DC infected versus uninfected groups (*p* = 0.3099).

#### Cancellous bone volume

Cancellous bone volume results are shown in Fig. 4 *E*. The median cancellous bone volume in the infected group receiving no current was 0.25 (range, 0.09 to 2.15) mm^3^, and was 1.23 (range, 0.44 to 2.22) mm^3^ in the 200 μA DC infected group (*p* = 0.0205). In the uninfected groups, the median cancellous bone volume of the group receiving no current was 0.13 (range, 0.10 to 0.32) mm^3^, and was 0.66 (range, 0.13 to 3.78) mm^3^ in the 200 μA DC group (*p* = 0.0031). When comparing the cancellous bone volume of the infected versus uninfected groups receiving no current, the infected group had significantly more volume than the uninfected group (*p* = 0.0109). There was no difference in trabecular separation when comparing the 200 μA DC infected versus uninfected groups (*p* = 0.5660).

#### Cancellous bone density

Cancellous bone density (BS/TV) results are shown in Fig. 4 *F*. The median cancellous bone density in the infected group receiving no current was 4.32 (range, 1.57 to 18.58) mm^2^/mm^3^, and was 4.41 (range, 1.69 to 18.78) mm^2^/mm^3^ in the 200 μA DC infected group (*p* = 0.7324). In the uninfected groups, the median cancellous bone density of the group receiving no current was 2.59 (range, 2.16 to 3.46) mm^2^/mm^3^, and was 6.27 (range, 2.51 to 17.58) mm^2^/mm^3^ in the 200 μA DC group (*p* = 0.0054). When comparing the cancellous bone density of the infected versus uninfected groups receiving no current, the infected group had significantly more density than the uninfected group (*p* = 0.0034). There was no difference in trabecular separation when comparing the 200 μA DC infected versus uninfected groups (*p* = 0.8253).

#### Strength testing

**Figure 5:**
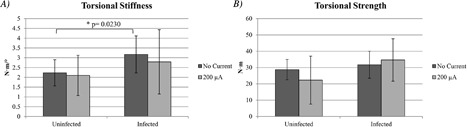
Mean with ± SD torsion measurement of infected and uninfected bones receiving 200 μA fixed direct electrical current or no current: (*A*) torsional stiffness and (*B*) torsional strength.

Torsional stiffness results are shown in Fig. 5 *A*. When testing the torsional stiffness of the infected group receiving no current, the median value was 3.09 (range, 1.25 to 4.44) *N* · m/degree, and was 2.53 (range 0.94 to 6.08) *N* · m/degree in the 200 μA DC infected group (*p* = 0.2184). In the uninfected groups, the median torsional stiffness was 2.35 (range, 0.67 to 2.87) *N* · m/degree in the group receiving no current and in the 200 μA DC group it was 1.95 (range, 0.5 to 3.43) *N* · m/degree (*p* = 0.4273). When comparing the torsional stiffness of the uninfected versus infected groups receiving no current, the infected group was stiffer (*p* = 0.0230). There was no significant difference between the 200 μA DC uninfected versus infected groups.

Torsional strength results are shown in Fig. 5 *B*. When testing the torsional strength of the infected group receiving no current, the median value was 33.81 (range, 14.83 to 41.40) *N* · m, and was 36.35 (range, 9.86 to 61.21) *N* · m in the 200 μA DC group (*p* = 0.4602). In the uninfected groups, the median torsional strength was 29.13 (range, 17.21 to 37.55) *N* · m in the group receiving no current and in the 200 μA DC group it was 18.51 (range, 8.10 to 51.05) *N* · m (*p* = 0.1530). There was no significant difference in torsional strength between the uninfected versus infected groups receiving no current or receiving 200 μA DC.

#### Pathology

**Figure 6:**
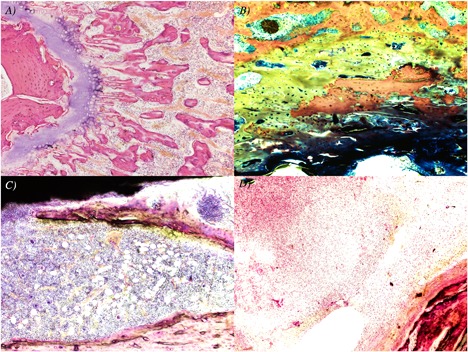
Histopathology of bones. Images are at ×100 magnification. (*A*) Uninfected, no current (H&E stain); (*B*) uninfected, 200 μA fixed direct electrical current (trichrome stain − osteoid is green); (*C*) infected, no current (H&E stain); and (*D*) infected, 200 μA fixed direct electrical current (H&E stain).

Representative histopathologic images are shown in Fig. 6. In the uninfected group receiving no current (Fig. 6 *A*), there was no inflammation and no remarkable bony changes/remodeling were noted. In the uninfected 200 μA DC group, one sample was not interpretable (possibly because of a poor cut); in the other bones, one of five had acute inflammation around the insertion point of the platinum electrode, with no apparent inflammation along the length of the electrode, and all five bones exhibited mild‐to‐moderate osteoid formation (Fig. 6 *B*).

In the infected group receiving no current, findings from two of the six bones were not interpretable because of poor cuts. For the remaining four bones, a mild‐to‐high degree of acute inflammation was present, mainly around the electrode, and there were no remarkable bony changes or findings of remodeling (Fig. 6 *C*). In the infected 200 μA DC group, a mild‐to‐moderate degree of inflammation was seen in all bones, and mild‐to‐moderate osteoid formation was noted (Fig. 6 *D*).

## Discussion

The μCT results showed an increase in the cortical bone volume and density as well as more trabecular thickness, cancellous bone volume, and density in the 200 μA DC group compared with the no current group in both the uninfected and infected bones, with the exception of cortical bone density in the infected bones. These results could be explained by the electrical current causing an increase in osteoid presence, as evidenced by the pathology results, leading to bone formation. The strength testing did not reveal any differences between no current and 200 μA DC between the infected and uninfected bones, but among the bones receiving no current, the infected group had more torsional stiffness than did the uninfected group. This observation has been noted in a study by Horst and colleagues, in which mice tibias were infected with *S. aureus* and 2‐months postinfection the bones were removed and underwent biomechanical testing. They noted that the infected tibias displayed increased torsional rigidity (torsional stiffness in our case) with almost a 30% decrease in flexibility, which supports a higher risk of fracture.^14^

Since the mid‐1950s, the effects of DC on healing bony non‐unions have been studied, and some evaluation of the effect of DC on bones has been performed.^15^, ^16^, ^17^, ^18^, ^19^, ^20^ Friedenberg and colleagues tested the effect of 1 to 100 μA on bone in a rabbit femur model, by placing a stainless steel anode and cathode connected to a power source and delivering current for 10 days. By means of microscopic findings, using 50 and 100 μA, they noted marked dark discoloration of the bone around the anode and cathode; tissue necrosis occurred mainly at the anode at these amperages, whereas bone formation occurred predominantly around the cathode using 5 to 20 μA.^17^ Although this study provides some insight as to what might be expected in bones exposed to low‐amperage DC, there are some key differences to our study, including the type of metal used in the anode. We used platinum and they used stainless steel. The dark discoloration they observed could have been from corrosion of the stainless steel cathode and anode, as we have seen this when using stainless steel electrodes in a rabbit model of foreign body osteomyelitis treated with 200 μA DC.^7^ Notably, we did not observe discoloration or any other signs of corrosion of the anode with the use of platinum. On pathological and μCT scans, we did not observe tissue necrosis, nor did bones lose torsional stiffness or strength. Another major difference is that we were able to use a high‐resolution μCT instrument to give us more accurate measurements of the bone components, as compared with using only microscopic findings.

A limitation of our study is that we only tested a single amperage (200 μA), using a single electrode composition, a single duration of treatment (14 days), and one strain of *S. epidermidis*. It would be valuable to test other amperages (lower and higher than 200 μA) and organisms in our model, as well as longer treatment times. We chose the amperage of 200 μA based on *in vitro* comparisons of 20‐, 200‐, and 2000‐μA DC.^9^, ^21^ In addition, it would be valuable to test other anodic and cathodic metal compositions, as these could have different effects on bone, either positive or negative.

Another limitation is when imaging the bones and testing strength, the implant remained in the bone as removal would have been difficult and caused breakage of the bones; this caused interference/scatter in the μCT instrument. To overcome this limitation, we defined a ROI to measure.

A further limitation of this study is that the effect of DC on bones was not tested in the presence of antimicrobials. We have previously shown that 200 μA DC alone was more efficacious than antimicrobial in an experimental *S. epidermidis* chronic foreign‐body osteomyelitis model.^7^ However, we did not test combination treatment in this study.^21^ More studies are necessary, including bone testing in the presence of an antimicrobial agent.

With the advent of antibiotic resistant bacteria and the challenge of biofilm‐associated resistance, a universal antibiofilm therapy, especially one that might allow implant retention, would be of great advantage in clinical practice. The concept of applying electrical antibiofilm strategies to orthopedic hardware that is already in place is appealing, so long as toxicity and adverse effects are minimized. This can likely be accomplished with an optimized electrical antibiofilm system.

The findings we present here show that low‐amperage fixed DC increases cortical bone volume and density, as well as cancellous bone volume and trabecular thickness, possibly because of the formation of new bone, but does not affect torsional strength or stiffness.

## Disclosures

Dr. Patel reports grants from CD Diagnostics, BioFire, Curetis, Merck, Contrafect, Hutchison Biofilm Medical Solutions, Accelerate Diagnostics, Allergan, EnBiotix, Contrafect and The Medicines Company. Dr. Patel is or has been a consultant to Curetis, Specific Technologies, Selux Dx, GenMark Diagnostics, PathoQuest, Heraeus Medical, and Qvella; monies are paid to Mayo Clinic. In addition, Dr. Patel has a patent on *Bordetella pertussis/parapertussis* PCR issued, a patent on a device/method for sonication with royalties paid by Samsung to Mayo Clinic, and a patent on an anti‐biofilm substance issued. Dr. Patel receives travel reimbursement from ASM and IDSA and an editor's stipend from ASM and IDSA, and honoraria from the NBME, Up‐to‐Date and the Infectious Diseases Board Review Course. All other authors have no disclosures.
